# Supplementary figures and images for: Quantification of ultrasonic texture intra-heterogeneity via volumetric stochastic modeling for tissue characterization
Source: Med Image Anal. 2015 Apr;21(1):59–71. doi: 10.1016/j.media.2014.12.004 (PMC4339203; doi:10.1016/j.media.2014.12.004)

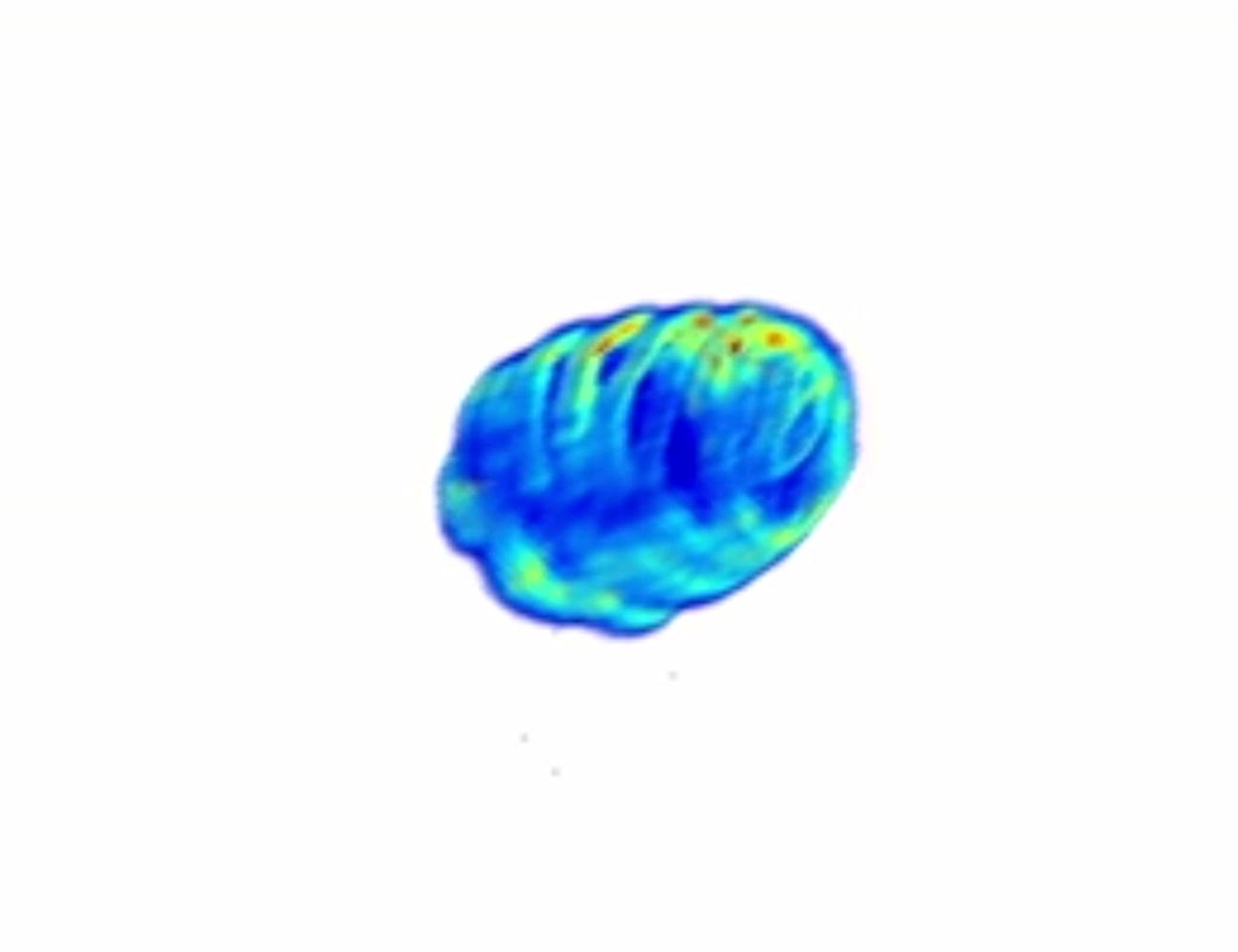

Supplement: Supplementary video 1 [file mmc1.jpg]

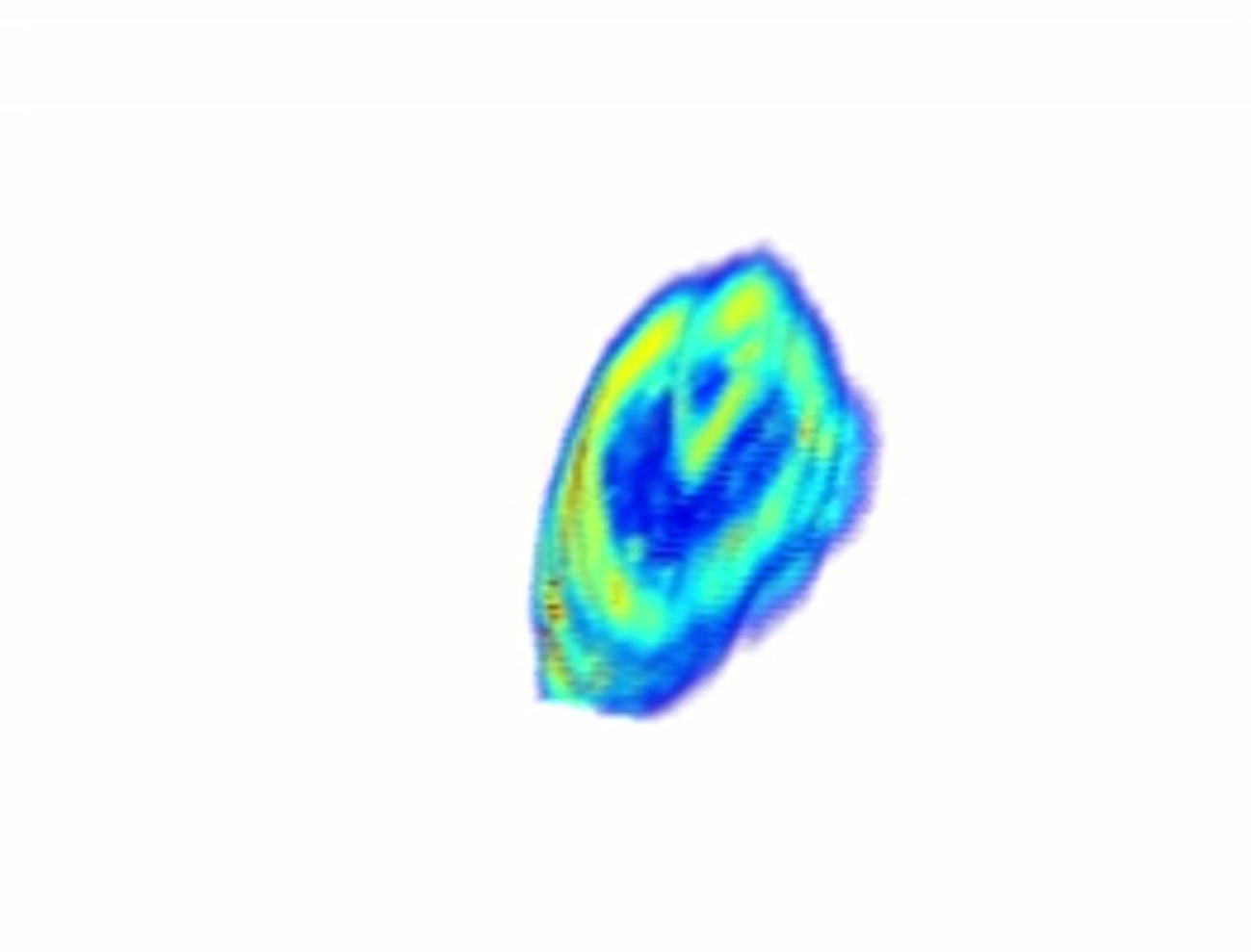

Supplement: Supplementary video 2 [file mmc2.jpg]

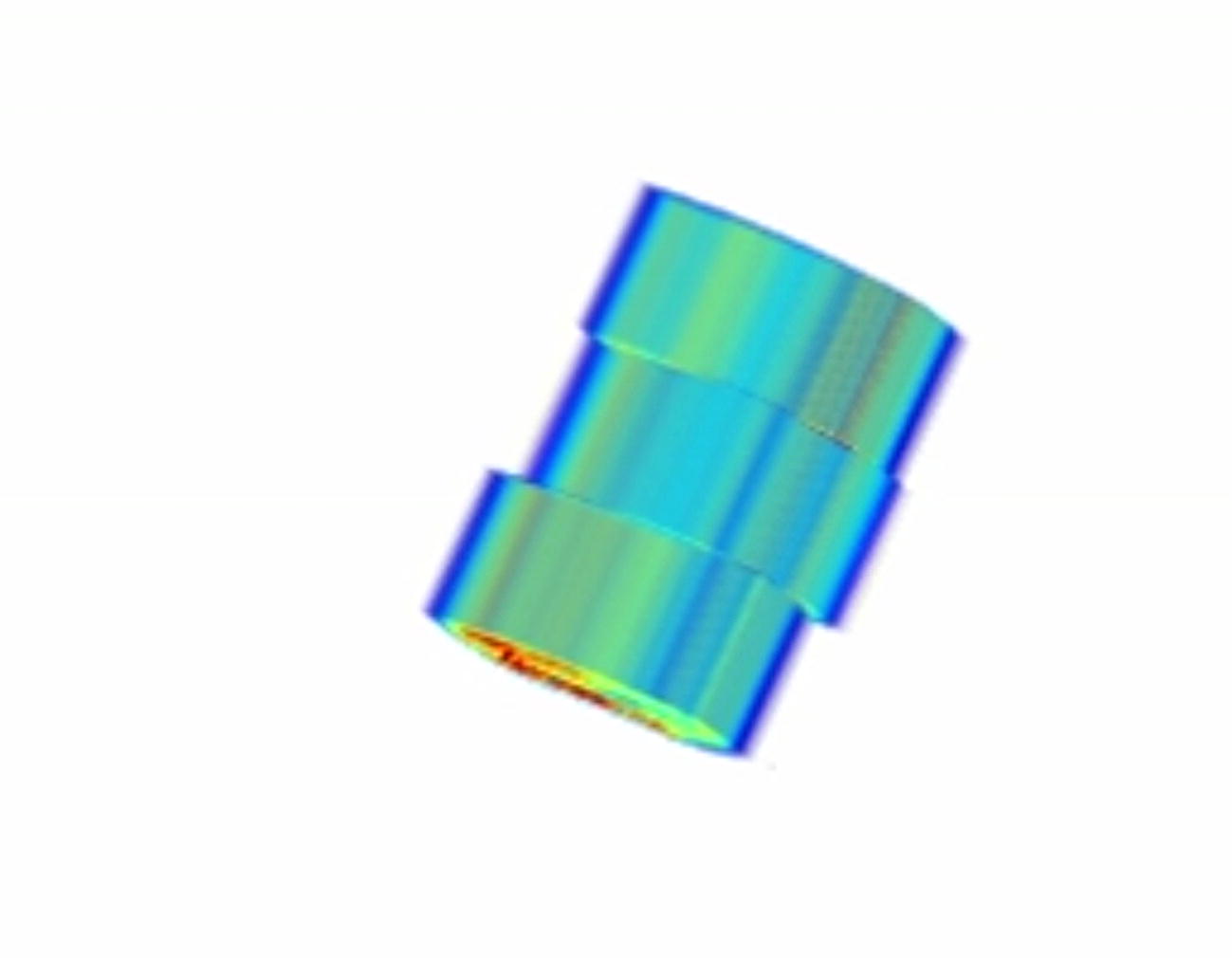

Supplement: Supplementary video 3 [file mmc3.jpg]

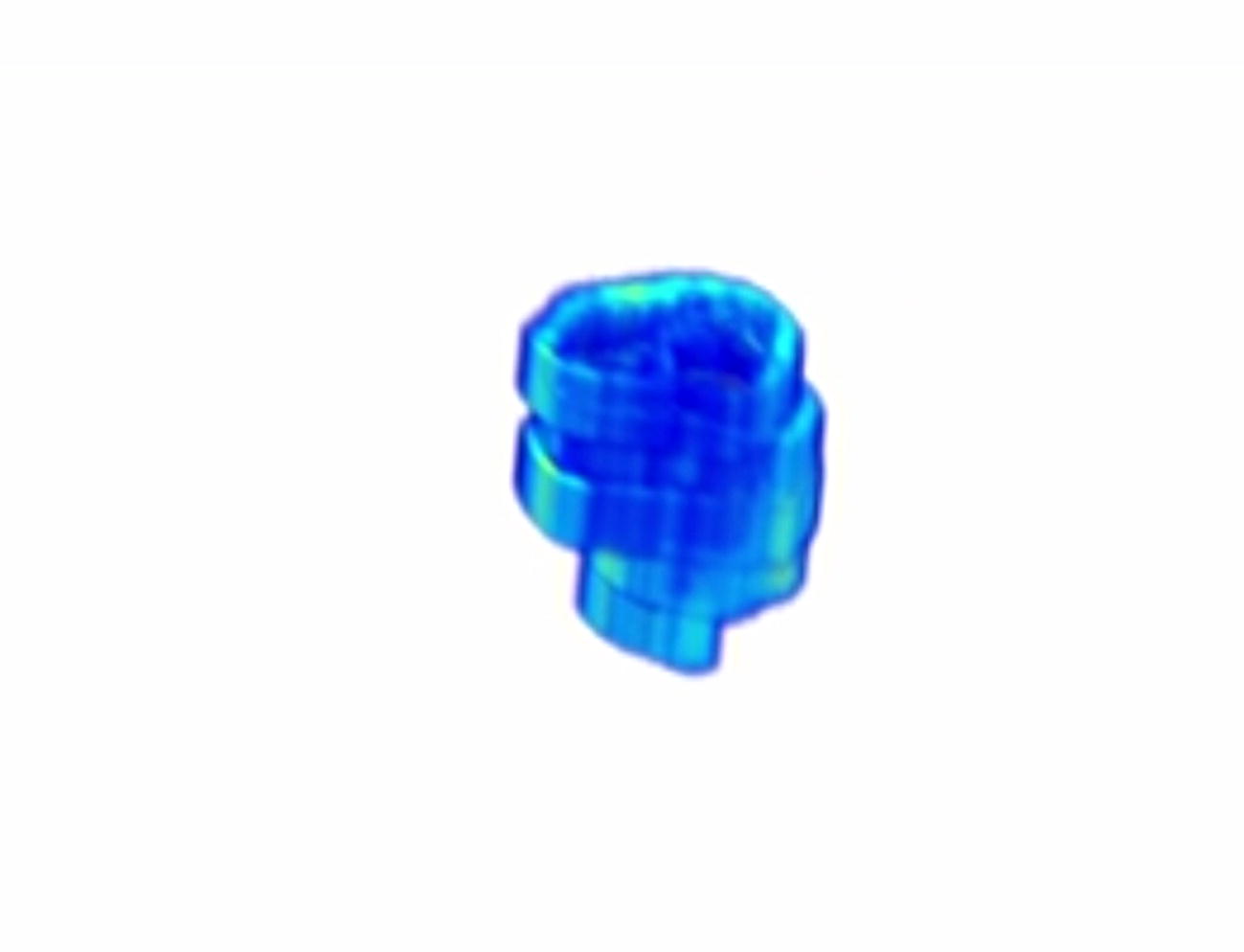

Supplement: Supplementary video 4 [file mmc4.jpg]
